# Region‐Based Segmentation of Lymph Node Metastases in Whole‐Slide Images of Colorectal Cancer: A Pilot Clinical Study

**DOI:** 10.1002/cam4.71449

**Published:** 2026-02-20

**Authors:** Alexey Fayzullin, Nikita Savelov, Artur Balkivskiy, Elena Ivanova, Anna Timakova, Vladimir Funtikov, Ekaterina Shelomentseva, Nikita Panchenko, Igor Spiridonov, Ivan Korkonishko, Mariia Makhina, Evgeniia Kutuzova, Daur Meretukov, Natalia Kretova, Tatiana Demura, Peter Timashev, Ruslan Parchiev

**Affiliations:** ^1^ Institute for Regenerative Medicine Sechenov University Moscow Russia; ^2^ Moscow City Oncology Hospital No. 62 Istra Russia; ^3^ Medical Neuronets Moscow Russia; ^4^ B.V. Petrovsky Russian Research Center of Surgery Moscow Russia; ^5^ Moscow Center for Healthcare Innovations Moscow Russia; ^6^ Institute of Clinical Morphology and Digital Pathology Sechenov University Moscow Russia; ^7^ Synapse Tech Moscow Russia

**Keywords:** artificial intelligence, colorectal cancer, computational pathology, computer vision, digital pathology

## Abstract

**Background:**

Digital technologies and artificial intelligence (AI) are transforming medical diagnostics, particularly in pathology. This study presented a two‐stage computer vision model designed to detect colorectal cancer metastases in whole slide images (WSIs) of lymph nodes.

**Methods:**

We developed a classification–segmentation pipeline optimized for both accuracy and efficiency. The model was trained on 108 WSIs and evaluated on 554 WSIs collected from two institutions using Leica Aperio AT2 and Hamamatsu NanoZoomer S360 scanners.

**Results:**

The classification model achieved a recall of 1.0 and a specificity of 0.935, while the segmentation model reported a Dice coefficient of 0.818 ± 0.105. Pathologists appreciated the model's precision in distinguishing solitary cancer cells from histiocytosis, reducing the need for peer consultations. Feedback from the pilot study indicated that the AI tool served as a valuable second opinion, enhancing diagnostic confidence.

**Conclusion:**

This study explored the practical applications of AI in clinical pathology, offering perspectives from both pathologists and data scientists. Our findings highlighted how AI can streamline workflows, improve diagnostic accuracy, and support personalized treatment planning. The integration of AI into pathology workflows has the potential to redefine diagnostic standards while maintaining the critical role of pathologists in decision‐making.

## 1 Introduction

Colorectal cancer is a type of malignancy that originates in the cells lining the colon or rectum, which are parts of the large intestine in the lower part of the body's digestive system. It is the third most common cancer globally, affecting both men and women, with an estimated 1.9 million new cases and 935,000 deaths in 2020 alone according to the World Health Organization [1].

The “Dataset for histopathological reporting of colorectal cancer” from The Royal College of Pathologists requires the excision of all regional lymph nodes and recommends comprehensive assessment of a minimum of 12 nodes for cancer staging [2]. This approach is integral for accurate prognostication, as the involvement of lymph nodes is a significant determinant of survival and influences postoperative treatment decisions [3, 4]. However, the extensive lymph node assessment places a considerable workload on pathologists, as each node needs to be meticulously examined microscopically to identify any potential metastatic involvement, which can be both time‐consuming and challenging due to the size and subtle changes of early metastases.

When examining lymph nodes from patients with colorectal cancer, pathologists scrutinize the size, shape and color of the lymph nodes and identify pathological changes such as the presence of singular tumor cells, often requiring immunohistochemical staining for precise identification. Metastatic colorectal cancer cells in lymph nodes are marked by distinct morphological features such as irregular size and shape, larger and hyperchromatic nuclei, increased cell division, disrupted tissue structures and elevated levels of cell death. Failure to identify lymph node metastases in colorectal cancer patients poses significant risks, including under‐staging of the disease which can lead to suboptimal treatment strategies [5, 6, 7, 8, 9].

The integration of artificial intelligence (AI) into the field of pathology revolutionizes the analysis of lymph nodes in patients with colorectal cancer, significantly reducing the time of laboratory investigation. Machine learning can enhance the sensitivity and specificity of lymph node assessment, thereby contributing to more precise staging and improved patient management strategies. Several studies addressed the challenge of lymph node metastases in colorectal cancer, initially focusing on predicting metastatic potential based on the primary tumor's histology [10, 11, 12, 13, 14]. This method, however, is not entirely reliable, as metastases can occur in cases where the primary tumor appears less aggressive. It is noteworthy that recent meta‐analysis showed no strong correlations between many histopathological features (lymphovascular invasion, lesion depth, necrosis, etc.) and lymph node metastasis status [15]. As a result, research has evolved towards the detection of lymph node regions exhibiting morphological features of metastasis [16, 17, 18]. The current limitations of the models include a decrease in accuracy at external datasets and occasional false‐negative results for micrometastases presenting the hardest cases for the pathologists [10, 14, 17].

The future of AI assistance in pathology lies in creating smooth and unobtrusive user experiences that seamlessly integrate with the pathologist's routine. This approach could transform the way pathologists work, allowing them to review more slides accurately within the same timeframe, and focus their expertise on more complex tasks, contributing to improved patient outcomes.

Our project is aimed at developing practice‐oriented solutions for medical practitioners that can be easily integrated into existing information systems. We used AI to detect colorectal cancer metastases, in order to optimize pathologists' workflow. To provide robust analysis of whole slide images (WSI) of colorectal cancer slides, we developed a classification–segmentation pipeline, validated it with datasets from two independent institutions and acquired feedback from the pathologists participating in a pilot study.

## 2 Methods

### 2.1 Dataset Preparation

To train the model, 108 histological slides of lymph nodes were collected at Moscow City Oncological Hospital No. 62 (Moscow, Russia). This set included both slides with metastases of colorectal cancer (91 positive samples) and slides without metastases (17 negative samples). Inclusion criteria: (1) patient age over 18 years; (2) confirmed diagnosis of colorectal cancer. Exclusion criteria: (1) presence of other diagnosed cancer diseases; (2) presence of artifacts in the image. The slides were scanned on a Hamamatsu NanoZoomer S360 in ×40 magnification mode. Tumor tissue was annotated in QuPath version 0.3.0 by three pathologists with at least 4 years of experience. The annotations for each slide were then validated by a senior pathologist with 14 years of practical experience. This dataset of 108 .ndpi WSI files was referred to as the training dataset. The dataset was split into training (*n* = 78), validation (*n* = 15) and test (*n* = 15) sets to ensure comprehensive training, robust validation and accurate evaluation of the model's performance (Table 1).

**TABLE 1:** Overview of dataset characteristics.

Dataset type	Total slides	Slides with cancer	Slides without cancer	Annotation type	Histological scanner
Training (Masks)	108	91	17	Expert annotation masks of tumor tissue in WSI	Hamamatsu NanoZoomer S360
Validation (Binary labels)	514	102	412	Binary label on presence or absence of metastases in WSI	Hamamatsu NanoZoomer S360
Additional validation (different WSI format)	40	40	0	Expert annotation masks of tumor tissue in WSI	Leica Aperio AT2

A validation dataset was utilized to further assess the classification model's performance under different conditions. This dataset was prospectively collected from the routine workload of Moscow City Oncological Hospital No. 62 (Moscow, Russia), adhering to the same rigorous selection criteria to ensure consistency with the previously described datasets. It consisted of 514 WSIs, each annotated with binary labels indicating either the presence or absence of colorectal cancer metastases. These slides were also scanned with a Hamamatsu NanoZoomer S360 at a magnification of ×40. There were no expert annotations of tumor regions or masks created for the WSIs of this dataset.

The validation dataset included 102 slides with metastases and 412 slides without tumor tissue. This distribution reflected a weekly workload of colorectal cancer slides at the Pathology Department and allowed for a comprehensive evaluation of the classification model's sensitivity and specificity. The high number of negative samples in the dataset provided a challenge for the classification model, testing its ability to correctly identify slides without metastases and decrease the number of false positive predictions. This setup was crucial for validating the practical utility of the classification in clinical settings, where the accuracy in identifying both positive and negative cases directly impacts patient outcomes and treatment planning.

Slides for an additional validation dataset were collected in a histological archive of Sechenov University (Moscow, Russia) following the same selection criteria. The purpose of evaluating slides in this dataset was to evaluate the accuracy of the model on files of different modalities. This dataset comprised 40 .svs WSIs of lymph nodes with metastases of colorectal cancer. 40 slides were scanned on a Leica Aperio AT2 in ×40 magnification mode. Tumor tissue was annotated by two pathologists with at least 2 years of experience in QuPath version 0.4.3. The annotations for each slide were then validated by a senior pathologist with 16 years of practical experience.

### 2.2 Classification Model Architecture and Training Process

The GoogleNet architecture was chosen for classification due to its ability to capture image patterns at different magnifications and rapidly obtain model inference. For the pretrained model, binary cross‐entropy (BCE) was used as a loss function. The initial learning rate was set to 1e‐4, which was automatically adapted during the training process using the Adam optimizer.

WSIs were downscaled by 2‐fold and then cut into 1024 × 1024 tiles with an overlap of 20%. Each tile was then assigned a region with a mask. If a pathologist's annotation in a given area was more than 50 pixels in size, the entire tile was marked as “tumor”. Testing took place on 15 slides not included in the training set.

Several augmentations were applied (Rotate, HorizontalFlip, VerticalFlip, RandomRotate90, Transpose, ElasticTransform, CoarseDropout, HueSaturationValue, RandomBrightnessContrast, RGBShift, Normalization) to increase the data sample by modifying existing images. GoogleNet was trained for 20 epochs with a batch size of 4.

To determine the optimal threshold for binary classification, we performed a grid search across a range of values from 0.0 to 1.0 in increments of 0.01 using the validation dataset. We evaluated performance using F1‐score, specificity and recall, and observed that while the optimal thresholds slightly varied across folds or experiments, the performance plateaued near the default threshold of 0.5. Therefore, to ensure consistency and avoid overfitting, we retained the threshold of 0.5, which yielded robust results without degrading per‐slide performance.

Some errors in classification were associated with artifacts in the form of tissue folds that formed during the preparation of material (particularly, during microtome sectioning) for histological examination. The neural network identified these artifacts as tumor areas. Since these artifacts had evident color patterns, we were able to filter them at the image preprocessing stage using a threshold on the values of the color channels. In order to prevent the errors occurring due to insufficient coloring of the tissue sections, the training dataset included 10 WSIs with insufficient coloring.

After training and re‐validation, accuracy, recall and specificity were calculated and the results were presented in the form of a confusion matrix.

### 2.3 Segmentation Model Architecture and Training Process

We used DeepLabV3+ architecture for segmentation of histological images for the following reasons: (1) it allows capturing more complex features and patterns in medical images; (2) it has high segmentation accuracy on small objects, which is important in medical segmentation tasks where isolated tumor foci and fine tissue characteristics can be important for correct diagnosis and treatment; (3) it is effective in resource‐limited settings, which is often the case in healthcare settings.

The loss function in the segmentation task was also performed by BCE with the Adam optimizer. The initial learning rate also took the value 1e‐4 and was automatically modified by the optimizer during the learning process.

To train the segmentation model, the slides were first randomly divided into training and test datasets, with 78 slides designated for training and 15 for testing. After this division, the slides were segmented into tiles of 2048 × 2048 pixels with an overlap of 20%. Augmentations (Rotate, HorizontalFlip, VerticalFlip, RandomRotate90, Transpose, ElasticTransform, GridDistortion, OpticalDistortion, CoarseDropout, CLAHE, RandomGamma, GaussianBlur, HueSaturationValue, RandomB rightnessContrast, Normalization) were also used to generate data for the train dataset. DeepLabV3+ was trained for 20 epochs with a batch size of 4.

The metastasis segmentation was quantified using 2 metrics: Matthews correlation coefficient (MCC) and Dice score. The MCC between the ground truth and segmented labels was calculated as follows Equation (1):

(1) $$\mathrm{MCC}=\frac{\left(\mathrm{TP}*\mathrm{TN}-\mathrm{FP}*\mathrm{FN}\right)}{\sqrt{\left(\mathrm{TP}+\mathrm{FP}\right)\left(\mathrm{TP}+\mathrm{FN}\right)\left(\mathrm{TN}+\mathrm{FP}\right)\left(\mathrm{TN}+\mathrm{FN}\right)}}$$

where TP is the number of true positives, TN is the number of true negatives, FP is the number of false positives, and FN is the number of false negatives. MCC ranges between 1 and þ1, where þ1 represents a perfect prediction, 0 an average random prediction, and 1 an inverse prediction, when compared with the ground truth.

The Dice score is used to evaluate the similarity between a predicted segmentation mask and the ground truth segmentation mask. The Dice score ranges from 0, indicating no overlap, to 1, indicating perfect overlap Equation (2).

(2) $$\text{Dice score}=\frac{2*\mathrm{TP}}{2*\mathrm{TP}+\mathrm{FN}+\mathrm{FP}}$$

where TP is the number of true positives, TN is the number of true negatives, FP is the number of false positives, and FN is the number of false negatives. Expected ranges of Dice score for good, acceptable and bad score could be > 0.8, 0.6–0.8 and < 0.6 respectively. For statistical analysis, a one‐sample Student's *t*‐test was used to assess whether the mean Dice coefficient is statistically significantly different from the threshold (0.7). The calculation was performed using the standard formula adjusted for the sample standard deviation. A two‐tailed *p*‐value was calculated based on the Student's distribution with (*n*−1) degrees of freedom. Python, along with the NumPy and SciPy libraries, was used for these calculations.

We calculated metrics from 30 .ndpi and 40 .svs WSI files with expert annotation masks that were not previously used in training. This was done to assess the model's performance on images it had not encountered before and to demonstrate its ability to generalize and perform under novel conditions.

### 2.4 Evaluation of Experts' Experience With the Model

We created an online project within the Axon medical information system (version 1.0; Synapse Tech, Moscow, Russia) which contained three cases with WSIs of lymph nodes from colorectal cancer patients. Case I included four WSIs with metastatic lymph nodes, serving as an introduction to the system interface. Case II presented 15 WSIs, with 8 containing metastases: 4 with small metastatic foci and 4 with more prominent metastases. This case did not include any model‐generated masks. Case III also featured 15 WSIs in a similar distribution (7 clear, 4 with small metastases and 4 with notable metastases). The samples in Case III were processed using a model that generated semi‐transparent overlaying masks on the tumor tissue.

Expert validation was conducted among pathologists with a minimum of 4 years of experience in handling operative materials, including cases involving colorectal cancer. Both pathologists were from the B.V. Petrovsky Russian Research Center of Surgery (Moscow, Russia). Each task was recorded on video, allowing for precise determination of the assessment time for each WSI. In all cases, the online system was accessed using Google Chrome (version 123.0.6312.105). Statistical analysis was carried out with GraphPad Prism v. 9.00 (GraphPad Software Inc.). Intergroup differences were analyzed using the Kruskal–Wallis test followed by Dunn's multiple comparison test. The results were presented as scatter plot graphs. The significant level of differences *p* was taken at the value < 0.05. No statistically significant differences were identified between traditional and AI‐supported evaluations.

## 3 Results

### 3.1 Classification Model Performance

**FIGURE 1:**
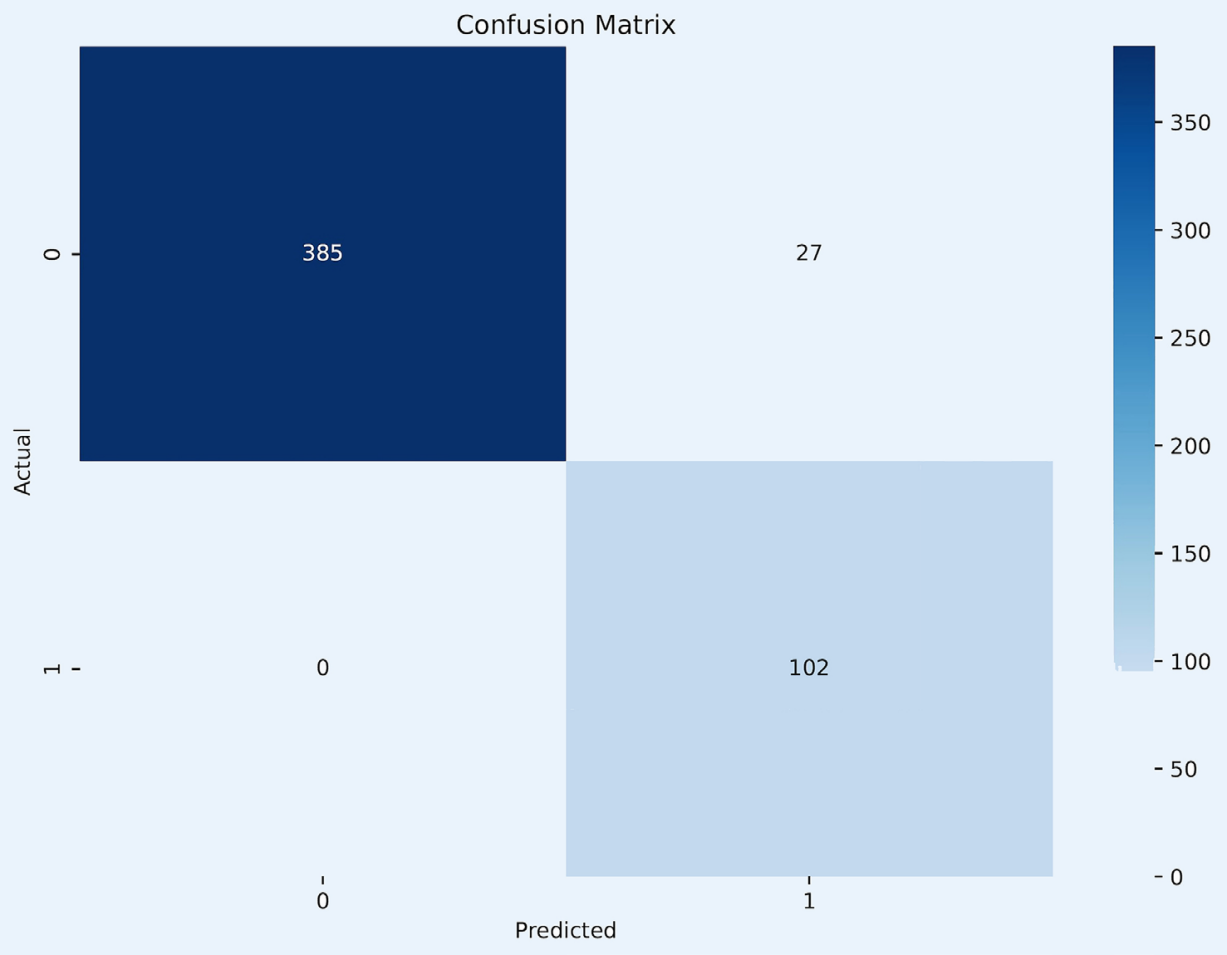
Confusion matrix of the classification model. The matrix visualizes the classification model's predictions compared to ground truth. The *x*‐axis represents the model's predicted labels, and the *y*‐axis shows the actual labels, illustrating the accuracy of the model across different scenarios. Label 0—no metastases, or “clean” lymph node; Label 1—presence of metastases, or metastatic lymph node.

The model classified all WSIs of lymph nodes with metastases correctly. However, it misclassified 27 negative cases as positive (Figure 1).

Classification accuracy = (102 + 385)/(102 + 0 + 385 + 27) = 94.75%

Classification recall = 102/(102 + 0) = 100%

Classification specificity = 385/(385 + 27) = 93.45%

**FIGURE 2:**
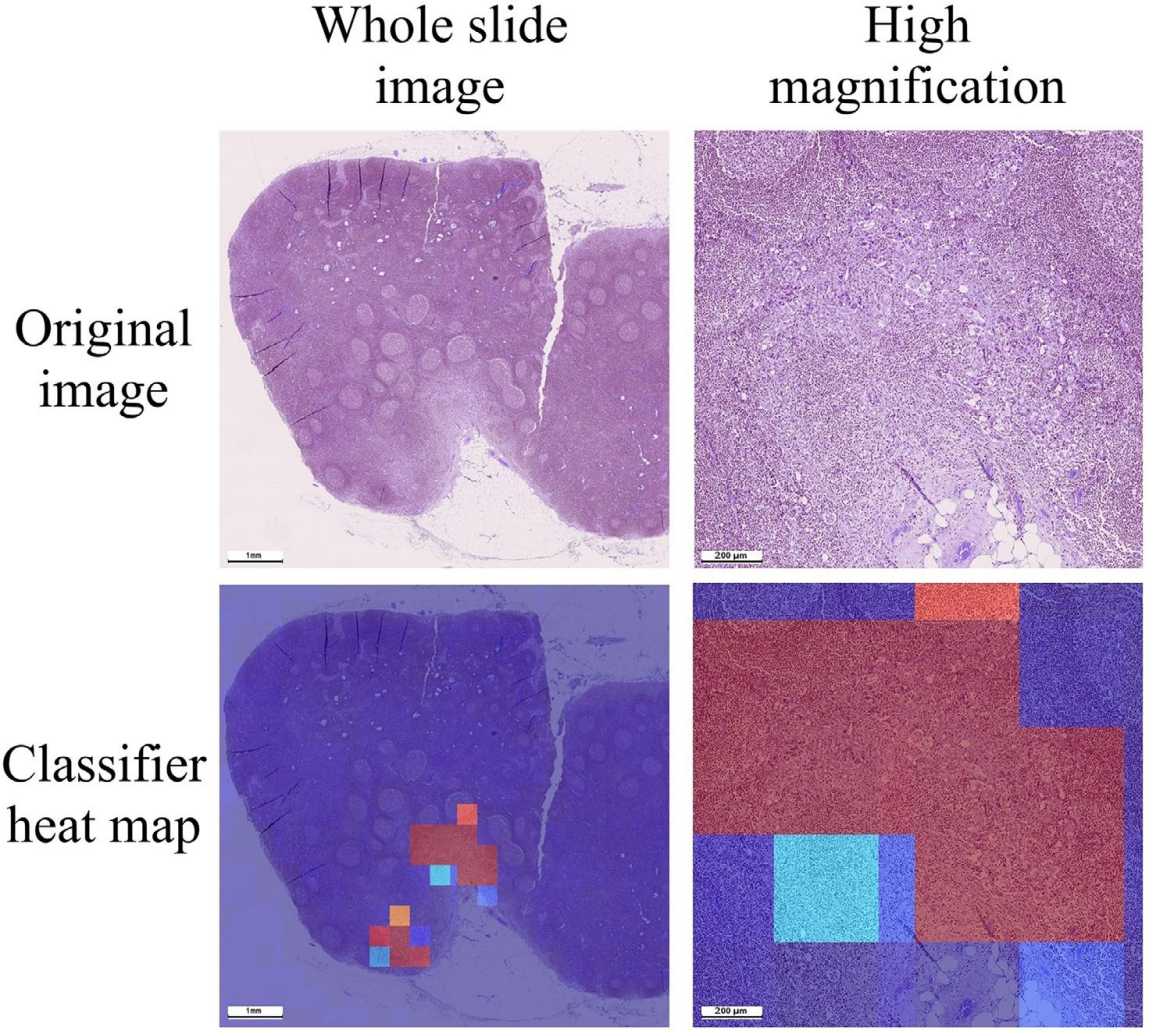
Heat‐map of results of the classification model. Blue color indicated regions classified as non‐suspicious and red color indicated regions classified as suspicious to the presence of metastases.

Classification model showed perfect performance with 100% recall and 93.45% specificity. Results of the classification model were visualized on heatmaps of whole slide images. The heatmaps helped to identify tumor regions and were sensitive to both small and large metastases. Classification model highlighted areas with both metastases and surrounding tissues which was useful for optimization of input data for segmentation model in the classification–segmentation pipeline (Figure 2).

### 3.2 Segmentation Performance

We evaluated the performance of our model and calculated the relevant metrics for 70 slides with metastases. Segmentation model MCC (Table 2) and Dice Scores (Table 3) were calculated for .svs and .ndpi WSIs separately and for a united dataset.

**TABLE 2:** MCC metrics of the segmentation model.

	.ndpi WSI dataset	.svs WSI dataset	United WSI dataset
Population_mean	0.7		
Sample mean	0.840	0.806	0.821
Sample std	0.086	0.103	0.097
Sample size	30	40	70
*t*_value	8.878	6.534	10.421
Degrees of freedom	29	39	69
*p*_value	9.1e‐10	9.4e‐08	8.3e‐16

**TABLE 3:** Dice metrics of the segmentation model.

	.ndpi WSI dataset	.svs WSI dataset	United WSI dataset
Population_mean	0.7		
Sample mean	0.841	0.801	0.818
Sample std	0.092	0.112	0.105
Sample size	30	40	70
*t*_value	8.444	5.695	9.415
Degrees of freedom	29	39	69
*p*_value	2.6e‐09	1.4e‐06	5.3e‐14

The obtained metrics let us perform statistical analyses to evaluate the performance of a classification model as part of a classification–segmentation pipeline.

Dice metrics of the segmentation model were 0.841 ± 0.092 for .ndpi dataset and 0.801 ± 0.112 for .svs which indicated only a limited loss of model effectiveness on files of different formats.

In both cases (MCC and Dice), we confidently rejected the null hypothesis, indicating that the segmentation model's performance is significantly better than “middle student” for these metrics.

The choice of specificity and Dice was driven by their relevance to the problem domain, their interpretability, and their role in balancing classification and segmentation tasks. The combined metric aimed to provide a comprehensive evaluation of the entire pipeline's performance by considering both tasks simultaneously.

### 3.3 Metastases Detection in Lymph Nodes

The region‐based approach combined the strengths of both classification and segmentation models allowing us to optimize speed and accuracy in processing WSIs. First, the images were segmented into tiles which underwent an initial assessment by the classification model. Tiles without signs of cancer were immediately discarded, streamlining the process by focusing only on potentially cancerous tiles.

**FIGURE 3:**
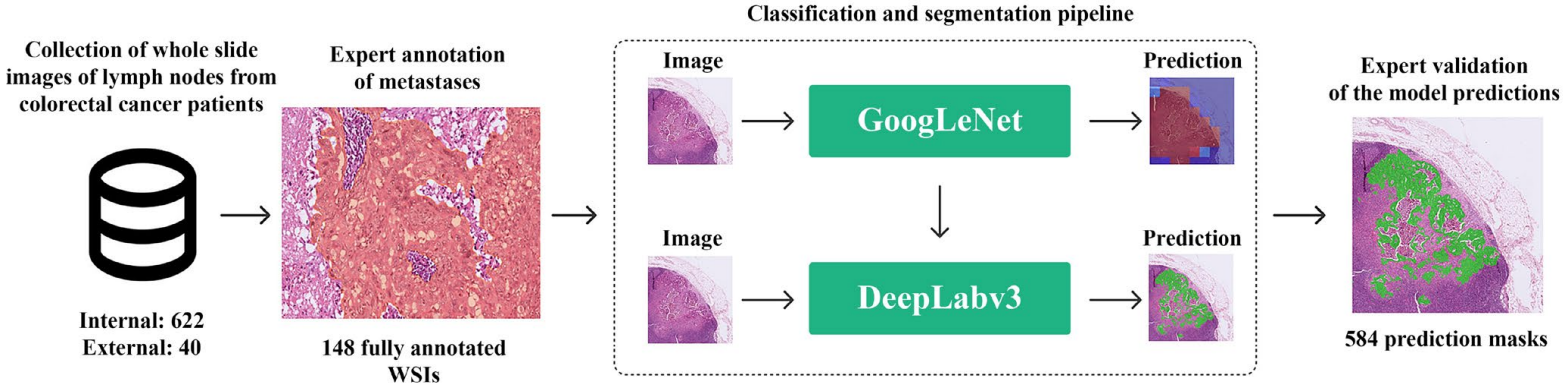
Study design. The classification–segmentation pipeline was trained on 78 annotated whole slide images using GoogleNet [19] and DeepLabV3_Resnet101 [20] architectures and tested on 584 WSI, including 70 slides with expert annotations of tumor regions.

Then, potentially cancerous tiles were processed through the segmentation model. This step involved grouping these tiles and applying the segmentation model to generate detailed masks. This not only aided in pinpointing the exact location and extent of tumor tissues but also ensured the accuracy of the diagnosis. In cases where the classification model detected cancer but the segmentation model did not confirm this, the final decision deferred to the segmentation model. This conservative approach prioritized accuracy over detection speed, minimizing false positive predictions. Semi‐transparent segmentation masks overlayed original H&E images and allowed assessing the precision of metastases predictions from the pathologist's perspective (Figure 3).

A minor portion of tiles (6.55% of the validation dataset) of normal lymph nodes without signs of metastases was misclassified as positive by the classification model. The classification model was sensitive to images with epithelial displacement, unfocused or significantly overstained regiones of germinal centers and medullar cords as well as rough artifacts of microtomy. These images were processed by the segmentation model and the generated masks highlighted epithelial displacement (usually on the edges of the section), unfocused germinal centers and microtomy artifacts including compact fragments of arteries and lymphoid tissue. However, the segmented false positive objects were small and did not include the whole area of the tissue structures making them subject to filtering without a significant decrease in the model sensitivity to the tumor cells. It is important to notice that the segmentation model did not generate false positive masks for the peritumoral stromal component of the lymph node, for sinus histiocytosis or for the staining artifacts.

**FIGURE 4:**
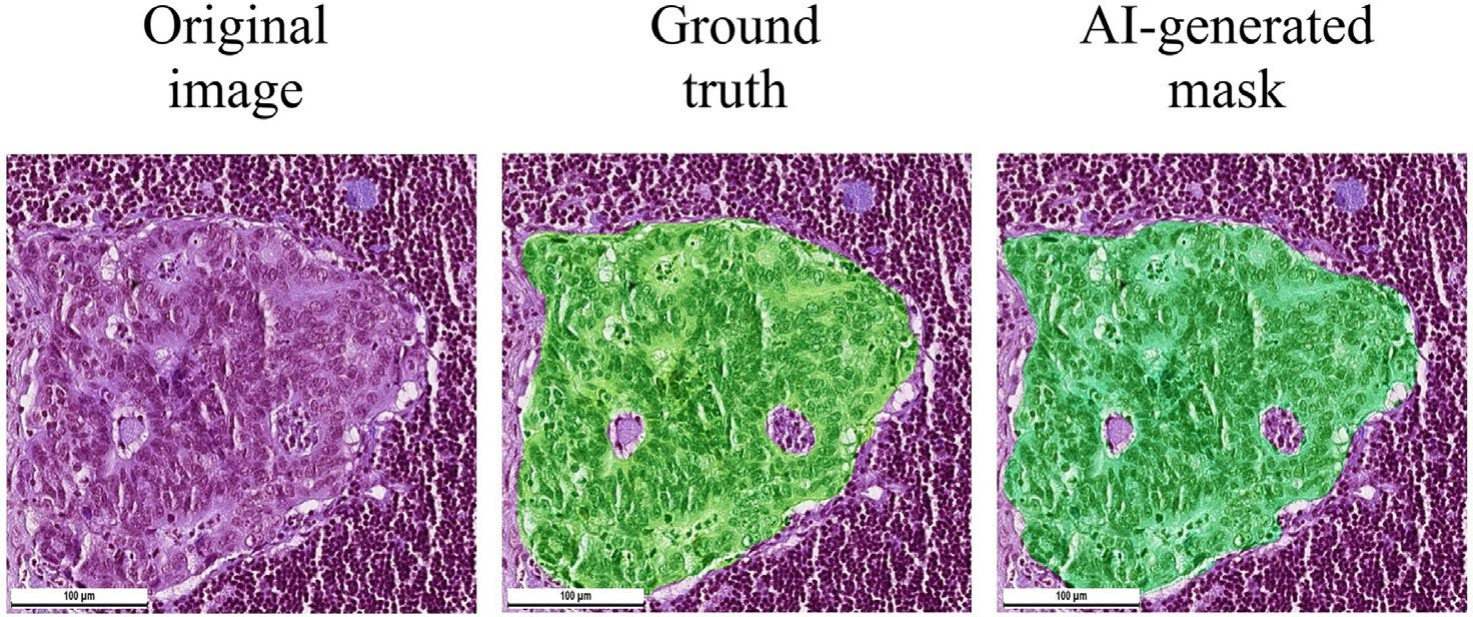
Comparison of expert's manual segmentation and AI‐generated mask. Segmentation model was trained on 78 annotated whole slide images using DeepLabV3_Resnet101 semantic segmentation model. We observed that AI‐generated masks better demarcated tumor cells from surrounding lymphoid tissue. The model did not highlight inclusions inside the tumor such as lakes of mucin and necrotic detritus.

In the cases of WSIs of lymph nodes with metastases, the masks overlayed the groups of tumor cells (Figure 4).

**FIGURE 5:**
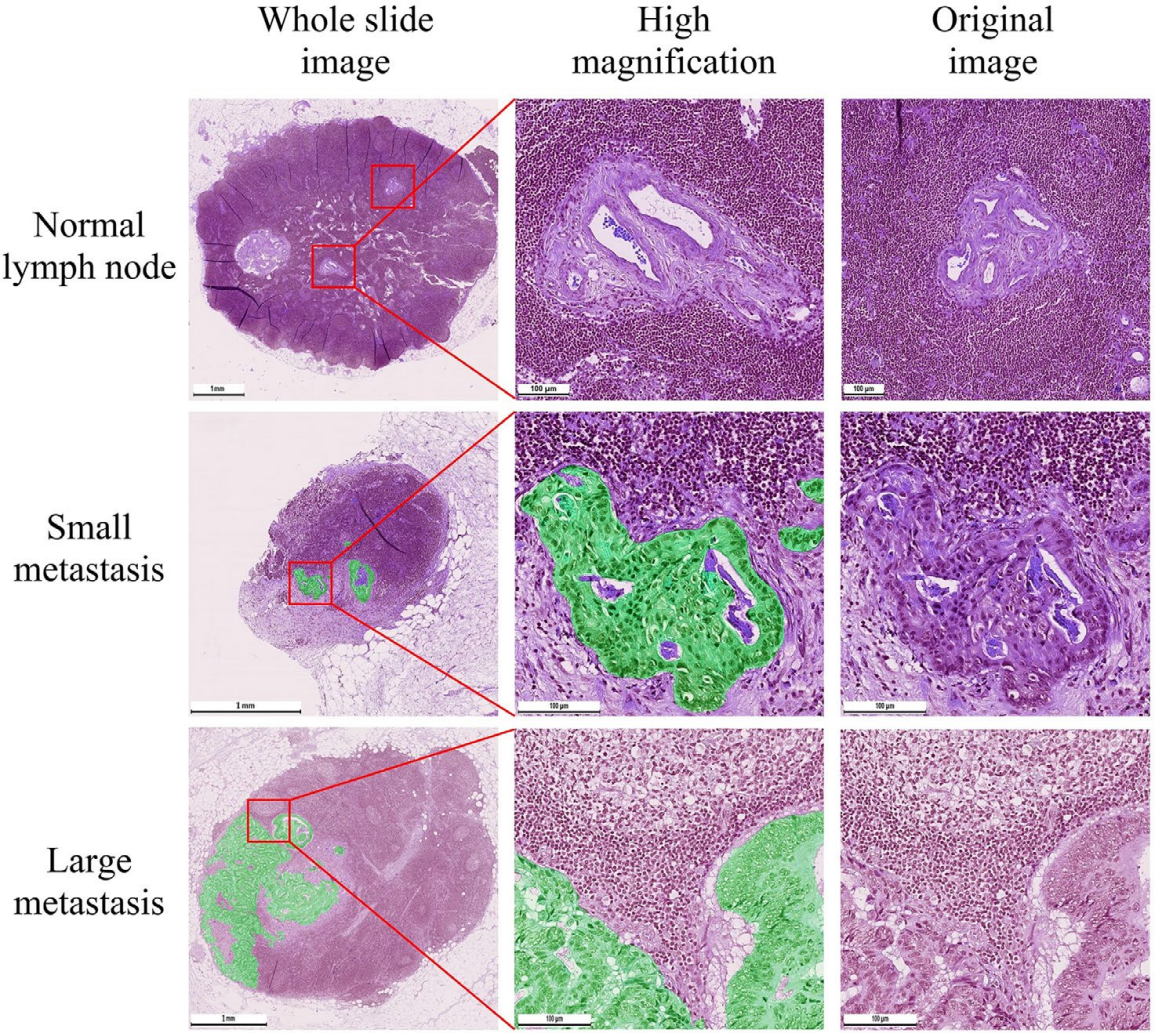
Segmentation of metastases in whole slide images of lymph nodes. The model generated an empty mask for normal lymph node (upper row). There were no regions segmented as tumor tissue in lymphoid tissue or lymph node stroma. It should be noted that histological artifacts such as folds did not cause false‐positive metastasis segmentation. Small regions of metastatic cells (middle row) were segmented by the model. The margins of the mask corresponded with the shape of tumor cells and did not include surrounding connective or lymphoid tissue. The model generated highly precise masks with geometries that are not possible to recreate manually by the ordinary annotation tools. Large regions of metastatic cells (bottom row) were highlighted by the model and corresponded with the shapes of tumor cells. The mask did not include mucin or necrotic detritus. It is important to notice that there was no false‐positive segmentation in the regions of sinus histiocytosis.

The masks highlighted the collectives of cells as single objects and provided an extremely accurate segmentation margin over the tumor cells. The mask excluded the findings that usually correspond with metastases such as lakes of mucin and necrotic detritus. The mask allowed for the assessment of the polymorphic shape of tumor cells that distinguish them from normal cells. The mask highlighted metastases that appeared both as glandular structures and as solid structures that consisted of atypical polymorphic prismatic cells. The mask allowed for easy evaluation of both the volume and the pattern of growth of the tumor. The model segmented tumor tissue in all of the WSIs of lymph nodes that contained metastases allowing for the calculation of a 100% recall (Figure 5).

### 3.4 Evaluation of Expert Work in the System

**FIGURE 6:**
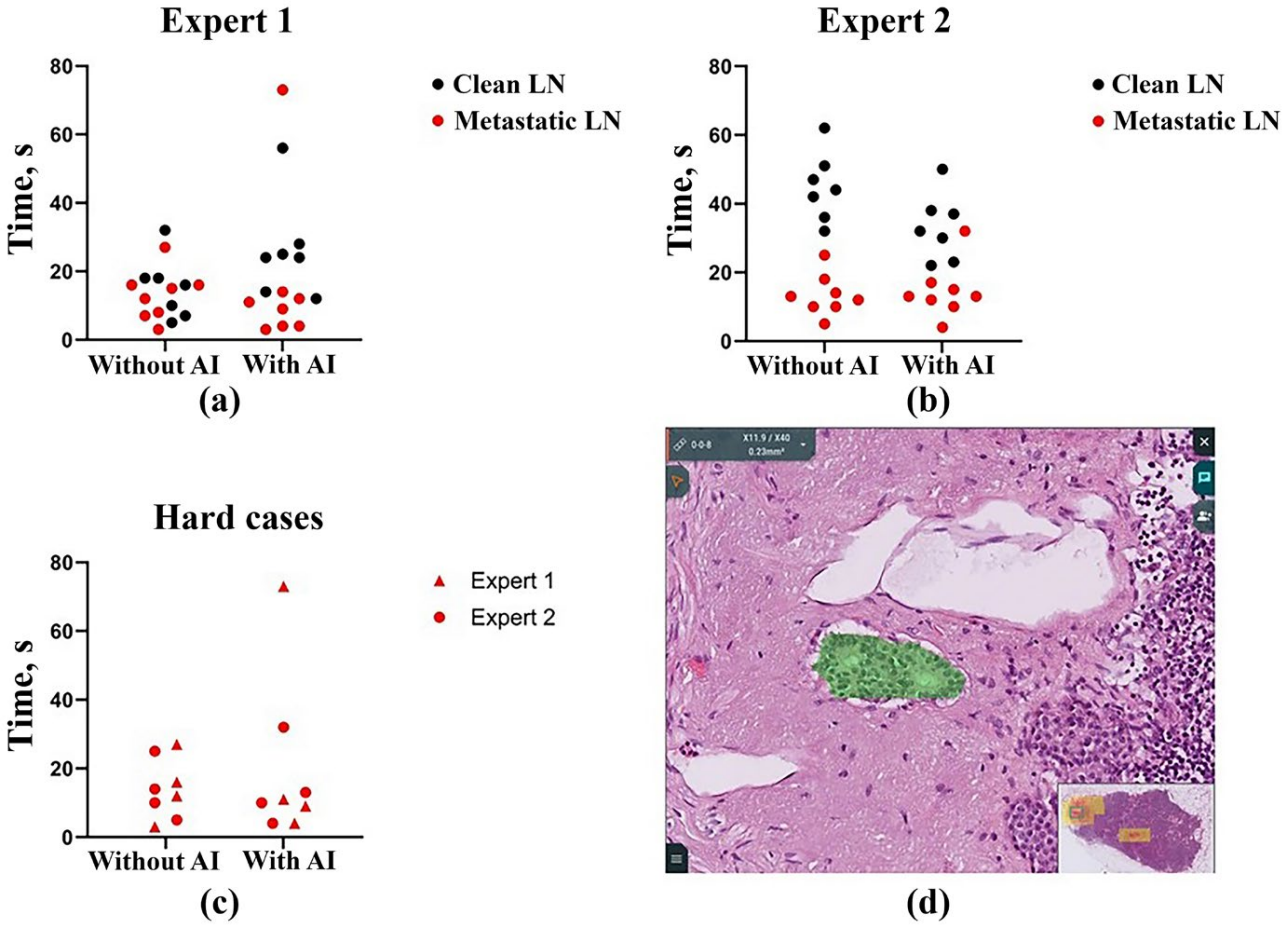
Evaluation of time spent on studying the whole slide images of lymph nodes. Segmentation mask on a solidary tumor metastasis (0.14 mm by 0.06 mm) that was identified as “easy to miss” on a screenshot from an online project in the Axon system. The metastasis had weak glandular features which made it challenging for the experts to distinguish it from the histiocytosis foci.

The time spent reviewing each scan by each expert was calculated (Figure 6). Expert 1 spent approximately the same amount of time on cases without metastases as on cases with metastases, while Expert 2 spent approximately twice as much time on cases with metastases. However, the speed of their work remained almost unchanged when using AI‐generated masks. The median time to complete the entire test task for pathologists decreased by 1 and 3 s, for Experts 1 and 2, respectively (15 s versus 14 s and 25 s versus 22 s). Moreover, if for Expert 1 this decrease occurred due to faster viewing of scans with metastases (10 s instead of 13.5 s), then for Expert 2 this happened due to a decrease in the time for assessing normal lymph nodes (32 s versus 44 s), and the time for assessing metastatic ones remained the same (13 s instead of 12.5 s). Separately, data were visualized for cases with small metastases, which pose the greater risk to be missed by the pathologist. In these cases, assessment times were also not significantly different. The median assessment time decreased by 2.5 s (10.5 s vs. 13 s). The results showed no statistically significant difference in time with or without AI assistance (*p* = 0.23 for Expert 1; *p* = 0.17 for Expert 2).

All pathologists paid attention to specimen 8 from case III, in which AI detected a micrometastasis (Figure 6D). They noticed that these areas were extremely “easy to miss,” as they resembled histiocytosis and had only minor signs of glandularity (similarity to glandular tissue), which characterize adenocarcinomas, including colorectal cancer. Identifying such areas may be a simpler task for AI than even for an experienced pathologist.

This pilot study showed that pathologists who used the Axon information system for the first time were able to effectively identify metastases in all histological specimens. The time to determine the lymph node status averaged 21.5 s, which may indicate that the race to speed up the work of the pathologist in this particular task may not be a priority and is inferior in importance to the accuracy of the diagnosis itself and the prevention of false‐negative conclusions.

The neural network distinguishes adenocarcinomas with weak signs of glandular tissue from normal tissue, which in some cases can save the doctor's time on consultation, and in others it can save against a false negative conclusion. Since such regions are poorly defined at low magnification, and the time for viewing a specimen “in routine” is 10–20 s, we propose that automatic detection of such regions is the most valuable result of the model. Feedback from pathologists was extremely positive, expressing a desire to implement such an AI assistant into their regular work.

## 4 Discussion

In this study, the region‐based segmentation model demonstrated high accuracy in detecting metastatic tumor cells in histological images of lymph nodes. We base the novelty and significance of our study results on the size of the sample, the unsupervised inclusion of the slides into the study, the inclusion of an external dataset prepared on a different scanner and pathologists' investigations into the precision of the generated masks. In this article, we provide strong evidence to support the classification–segmentation pipeline approach to the problem of tumor tissue segmentation.

The detection of metastases in WSIs of the lymph nodes is a focus of modern digital pathology. Chuang et al. reported training the AI model on unlabeled WSIs using ResNet‐50 on a supercomputer. The major goal of the study was the identification of micrometastases with a size between 0.2 and 2.0 mm and included single node cases. Macro‐ and micrometastases were accurately identified with an AUROC of 0.9944 and 0.9476, respectively. The class activation mapping revealed that the model focused on foci of metastatic cells exclusively ignoring areas of necrosis and mucin [16]. In our study, we specifically designed the pilot online project for expert evaluation to focus on macro‐ and micrometastases. We demonstrated that the model highlighted isolated tumor cells as small as 0.14 mm by 0.06 mm.

A recent publication reported an application of transfer learning to use data on breast cancer metastases to colorectal cancer cases. It was fine‐tuned with colorectal cancer annotations and showed up to 100% accuracy in detecting regions of lymph nodes with metastases. However, the absence of a tumor segmentation model limits it to detecting larger tissue regions, allowing the model to miss small isles of tumor cells. Because this approach was limited to a classification task, it was not possible to accurately measure the diameter of metastatic regions in lymph nodes. It is important to note that the authors reported the creation of an ensemble of CNNs, the former one—for the segmentation of lymph node tissue, the latter—a detector of tiles with metastases [17]. We solved this problem with a classification–segmentation pipeline based on GoogleNet for classification and DeepLabV3+ for segmentation. The latter architecture has high segmentation accuracy on small objects, which is important in medical segmentation tasks where small tumors or other objects can be critical for correct diagnosis and treatment.

Recently, a breakthrough clinical study investigated the efficacy of an AI‐assisted pathologist workflow. The group of researchers from Sweden developed a model trained on WSIs from their department to pixel‐level accuracy of 0.952 on nodes with metastases and 99% on a sample of 103 *N*+ lymph nodes. The major advantage of the study was that it is the first to report how much faster the AI‐assisted diagnostics are compared to traditional routine practice. The median time needed for evaluation was significantly shortened for at least one pathologist (median time for Pathologist 1, 26 vs. 14 s; median time for Pathologist 2, 25 vs. 23 s). This decision support tool has the potential to improve the diagnostic workflow by shortening the time needed for the evaluation of lymph nodes in colorectal cancer specimens without impairing diagnostic accuracy [18].

Interestingly, our pilot study showed that the time advantage can be absent for cases not requiring consultation. However, the feedback from pathologists was focused on a general positive reaction to support from AI and its ability to distinguish solitary tumor foci from histiocytosis. Possibly, the time‐saving effect can be more evident in cases of less‐experienced pathologists. If we advance the concept of a trusted AI system, we can speculate that a model with 100% sensitivity could assume the workload of analyzing non‐metastatic lymph nodes, significantly reducing the number of slides a pathologist needs to review per clinical case by a factor of 10. This approach does pose a risk, as the pathologist would still bear full responsibility for any under‐diagnosis, necessitating professional discourse. However, this scenario provides an opportunity for a controlled study, for example, by two independent groups of clinicians. Such a study could provide robust evidence either supporting the integration of AI in pathology or highlighting unforeseen consequences of shifting diagnostic responsibilities away from human pathologists.

## 5 Conclusion

The pilot test confirmed the hypothesis that the neural network's accuracy in determining the presence or absence of colorectal cancer metastases in lymph nodes is comparable to that of a pathologist's assessment. In some cases, the neural network identified isolated tumor cells that doctors had noted as “easy to miss”. These findings align with the broader investigation, in which a region‐based segmentation model consistently demonstrated robust performance in delineating metastatic foci. The strength of this approach rests on a heterogeneously sourced sample set, unbiased slide inclusion, validation on an external cohort and detailed expert review of the generated masks. Collectively, these elements reinforce the value of a classification‐segmentation pipeline as a reliable framework for tumor‐tissue detection and mapping in whole‐slide images.

## Author Contributions

**Alexey Fayzullin:** conceptualization, formal analysis, validation, data curation, investigation, writing – original draft, visualization. **Nikita Savelov:** supervision, writing – review and editing, validation, resources, conceptualization, investigation. **Artur Balkivskiy:** methodology, software, data curation, visualization, writing – original draft, investigation. **Elena Ivanova:** methodology, validation, investigation, writing – original draft, visualization. **Anna Timakova:** methodology, investigation, validation, writing – original draft. **Vladimir Funtikov:** writing – review and editing, software, investigation. **Ekaterina Shelomentseva:** software, writing – review and editing, investigation. **Nikita Panchenko:** software, writing – review and editing, investigation. **Igor Spiridonov:** investigation, writing – review and editing, software. **Ivan Korkonishko:** software, writing – review and editing, investigation. **Mariia Makhina:** conceptualization, investigation, writing – review and editing. **Evgeniia Kutuzova:** conceptualization, investigation, writing – review and editing. **Daur Meretukov:** conceptualization, investigation, writing – review and editing. **Natalia Kretova:** validation, writing – review and editing, investigation. **Tatiana Demura:** supervision, resources, validation, investigation, writing – review and editing. **Peter Timashev:** resources, investigation, writing – review and editing, project administration. **Ruslan Parchiev:** funding acquisition, resources, supervision, visualization, investigation, software, conceptualization, methodology, formal analysis, data curation.

## Funding

This work was supported by the Ministry of Science and Higher Education of the Russian Federation under grant agreement no. 075‐15‐2024‐640 (Sechenov University).

## Ethics Statement

All experimental protocols were approved by Moscow City Oncological Hospital No. 62 (Moscow, Russia).

## Consent

Informed consent was obtained from all subjects and/or their legal guardian(s) prior to having their data used in this study.

## Conflicts of Interest

Artur Balkivskiy, Vladimir Funtikov, Ekaterina Shelomentseva, Nikita Panchenko, Igor Spiridonov, Ivan Korkonishko, and Ruslan Parchiev have received funding for their work through employment at Medical Neuronets. Ruslan Parchiev has also received funding for his work through employment at Synapse Tech. All the other authors declare no conflicts of interest.

## Data Availability

The datasets generated during and/or analyzed during the current study are available from the corresponding author on reasonable request.
